# *PHOX2B* Tyr14Ter Mutation Might Be Associated with Sustained Diurnal Hypertension: Case Report and Review of the Literature

**DOI:** 10.3390/children13030425

**Published:** 2026-03-19

**Authors:** Fabio Antonelli, Simona Sottili, Maria Giovanna Paglietti, Alessandro Onofri, Renato Cutrera, Martina Mazzoni, Alessandro Rossi, Pierluigi Vuilleumier, Annalisa Allegorico

**Affiliations:** 1 Unit of Pediatric Pneumology and UTSIR, AORN Santobono-Pausilipon Children’s Hospital, 80129 Naples, Italy; fabantonelli65@gmail.com (F.A.); a.rossi@santobonopausilipon.it (A.R.); p.vuilleumier@santobonopausilipon.it (P.V.); 2 Department of Pediatrics, Emergency University of Bari, 70126 Bari, Italy; simona.sottili@hotmail.it; 3 Pediatric Pulmonology & Cystic Fibrosis Unit, Bambino Gesù Children’s Hospital, IRCCS, 00165 Rome, Italy; mgiovanna.paglietti@opbg.net (M.G.P.); alessandro.onofri@opbg.net (A.O.); renato.cutrera@opbg.net (R.C.); 4 Department of Medicine, Surgery and Dentistry “Scuola Medica Salernitana”, Pediatrics Section, University of Salerno, 84081 Baronissi, Italy; martmazz94@gmail.com

**Keywords:** *PHOX2B* mutation, CCHS, autonomic dysfunction, pediatric hypertension

## Abstract

**Highlights:**

**What are the main findings?**

- Non-polyalanine repeat mutations (NPARMs), including nonsense variants, are rare and generally associated with severe clinical phenotypes; however, the p.Tyr14Ter mutation may allow the production of a partially functional truncated protein, resulting in a relatively mild presentation.
- Baroreflex dysfunction in CCHS can manifest as orthostatic hypotension due to an inadequate blood pressure response to orthostatic stress and as impaired nocturnal vagal control leading to nocturnal hypertension.

**What are the implications of the main findings?**

- The genotype influences the prevalence of hypertension in children with CCHS, with a higher prevalence in patients carrying severe polyalanine repeat mutations (PARMs) compared with non-PARM genotypes.
- The presence of blood pressure abnormalities in a patient with an NPARM genotype, unlike previous reports, suggests that cardiovascular dysregulation may also occur in non-PARM CCHS.

**Abstract:**

**Introduction:** Congenital central hypoventilation syndrome (CCHS) is a rare disorder characterized by an impaired ventilatory response to hypercapnia and hypoxia, particularly during sleep, and frequently associated with autonomic dysfunction. It is caused by pathogenic variants in the *PHOX2B* gene. Although CCHS is typically diagnosed in the neonatal period, milder forms may present later in infancy or childhood, often triggered by respiratory infections. **Case presentation:** We report the case of 16-month-old male diagnosed with CCHS following an episode of hypoxemic–hypercapnic respiratory failure during respiratory syncytial virus (RSV) infection. His medical history included neonatal respiratory distress requiring oxygen therapy and recurrent wheezing. At 15 months, he developed acute respiratory distress with severe hypercapnia (PaCO_2_ 70 mmHg), requiring admission to the Pediatric Intensive Care Unit and invasive mechanical ventilation. Persistent sleep-related hypercapnia and hypoxemia prompted evaluation for central hypoventilation, confirmed by means of transcutaneous capnography and nocturnal pulse oximetry. Genetic testing revealed a de novo nonsense mutation in exon 1 of *PHOX2B* (p.Tyr14Ter). Brain magnetic resonance imaging showed diffuse white matter changes suggestive of gliosis. Further investigations identified early-onset systemic hypertension, requiring antihypertensive therapy. The patient was discharged on nocturnal non-invasive ventilation and enrolled in a neurodevelopmental rehabilitation program. **Conclusions:** This case highlights the phenotypic variability of CCHS and the importance of considering this diagnosis in children presenting with unexplained hypercapnia and sleep-related hypoxemia. It underscores the need for comprehensive autonomic evaluation, including blood pressure monitoring. The p.Tyr14Ter variant may allow partial protein function, potentially accounting for the relatively mild phenotype.

## 1. Introduction

Congenital central hypoventilation syndrome (CCHS) is a rare disorder of autonomic respiratory control, characterized by an impaired ventilatory response to hypercapnia and hypoxia, predominantly during sleep. It is caused by pathogenic variants in the *PHOX2B* gene, a critical transcription factor in the development of the autonomic nervous system. CCHS typically presents in the neonatal period with severe respiratory failure; however, milder phenotypes may manifest later in infancy or childhood, often triggered by respiratory infections that increase ventilatory demand and unmask underlying central hypoventilation. CCHS is increasingly recognized as a multisystem autonomic disorder. In addition to ventilatory impairment, affected individuals may exhibit abnormalities in the regulation of the cardiovascular, gastrointestinal, and neuroendocrine systems. Cardiovascular involvement has received particular attention due to evidence of impaired baroreflex function, which may result in orthostatic hypotension, loss of physiological nocturnal blood pressure dipping, and systemic hypertension [1]. These manifestations highlight the need for systematic autonomic evaluation, even in patients with apparently mild respiratory phenotypes. While most *PHOX2B* mutations are polyalanine repeat expansions (PARMs), non-polyalanine repeat mutations (NPARMs), including nonsense variants, are less common and are generally associated with more severe clinical presentations [2]. However, emerging evidence suggests that early truncating mutations may allow alternative translation initiation, leading to production of partially functional proteins and unexpectedly mild phenotypes, thereby complicating genotype–phenotype correlations [3]. Herein, we report a case of late-onset CCHS caused by a de novo *PHOX2B* nonsense mutation. The patient presented with sleep-related hypoventilation and early-onset systemic hypertension. This case highlights the phenotypic heterogeneity of CCHS and emphasizes the importance of early recognition and comprehensive autonomic assessment in children presenting with unexplained hypercapnia.

## 2. Case Presentation

**Figure 1:**
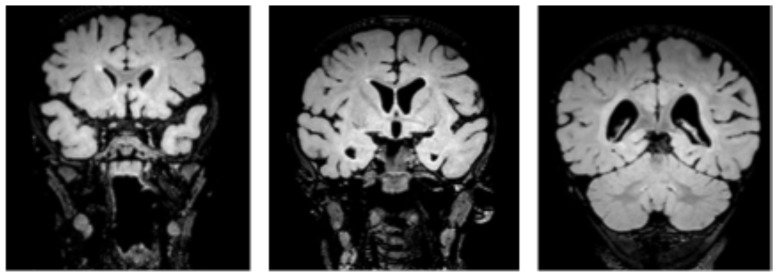
Brain MRI scan of the patient showing a quantitative reduction in bihemispheric white matter, particularly in the posterior regions, with T2/FLAIR hyperintensity observed in the corona radiata and supratrigonal/peritrigonal areas, partly associated with mild ectasia of the perivascular spaces, possibly due to gliosis. The supratentorial ventricular system appears larger than normal, with asymmetric lateral ventricles due to a greater size of the left ventricle and predominance of the posterior sections.

The case of a 16-month-old patient with congenital central hypoventilation syndrome (CCHS) who presented with persistent hypercapnia following an episode of hypoxic–hypercapnic respiratory failure during respiratory syncytial virus (RSV) infection is described. The patient was born at term (39 weeks of gestation) via uncomplicated vaginal delivery, following an uneventful pregnancy. At birth, somatometric parameters were appropriate for gestational age, and the APGAR score was 8 at both 1 and 5 min. Due to cyanosis and mild respiratory distress, the patient required high-flow oxygen therapy for ten days, followed by low-flow oxygen therapy for an additional two days. Due to suspicion of early-onset sepsis (EOS), supported by elevated C-reactive protein (CRP) levels, the patient received broad-spectrum antibiotic therapy, which resulted in normalization of the inflammatory markers. Blood cultures were negative. A neonatal cranial ultrasound was unremarkable, and the patient was discharged in stable condition. His past medical history revealed episodes of wheezing during upper respiratory tract infections, occurring approximately every 4 months. At 15 months of age, he was admitted to the emergency department at Santobono-Pausilipon Hospital, presenting with cough, feeding refusal, and dyspnea. On examination, he appeared tachypneic with subcostal retractions and an oxygen saturation (SpO_2_) of 85% on room air. Chest auscultation revealed bilaterally reduced breath sounds, fine crackles, occasional wheezing, and prolonged expiration. The patient received oxygen therapy via a Venturi mask (12 L/min, FiO_2_ 60%). Blood tests revealed neutrophilic leukocytosis with elevated inflammatory markers. PCR testing of a nasopharyngeal swab for respiratory pathogens was positive for RSV. Arterial blood gas analysis revealed respiratory acidosis [pH 7.33, partial pressure of arterial carbon dioxide (PaCO_2_) 70 mmHg]. Due to clinical deterioration, the patient was transferred to the Pediatric Intensive Care Unit (PICU), where he was intubated and started on invasive mechanical ventilation. The patient exhibited persistent hypercapnia, with PaCO_2_ levels remaining above 60 mmHg during sleep and decreasing during wakefulness. Once clinically stable, the patient was transferred to the Pulmonology Unit for further diagnostic evaluation. During sleep, transcutaneous (tc) CO_2_ monitoring was performed while on spontaneous ventilation, revealing tcCO_2_ values ≥50 mmHg for 97% of total sleep time, with an average of 58.3 mmHg. According to the American Academy of Sleep Medicine (AASM) criteria for nocturnal hypoventilation—which defines it as spending more than 25% of total sleep time with a tcCO_2_ level above 50 mmHg [4]—a diagnosis of nocturnal hypoventilation was made. Attempts at polygraphy on spontaneous ventilation failed due to hypoxia and worsening hypercapnia. CCHS was suspected, and assisted-controlled volume-targeted non-invasive nocturnal ventilation via nasal mask was initiated, resulting in rapid improvement of the patient’s overnight tcCO_2_ and SpO_2_ values. During recovery, ventilator settings were progressively adjusted to optimize oxygen saturation (SpO_2_) and gas exchange. To guide this process, multiple overnight pulse oximetry and transcutaneous capnography assessments were performed. Before discharge, capnography on non-invasive ventilation showed tcCO_2_ values ≥50 mmHg during only 8% of total sleep time, with an average of 41 mmHg. The patient was discharged after a 60-day hospital stay on assisted-controlled volume-targeted non-invasive nocturnal ventilation via nasal mask, to be used exclusively during sleep. Genomic DNA was extracted from peripheral blood. Targeted next-generation sequencing (NGS) was performed using a clinical exome panel that included genes associated with congenital central hypoventilation syndrome. Genetic analysis revealed a heterozygous nonsense mutation (c.42C>A, p.Tyr14Ter) in exon 1 of the *PHOX2B* gene, resulting in a premature stop codon at position 14. This variant was confirmed by means of Sanger sequencing. Parental testing was performed using the same approach and, as the mutation was not detected in either parent, it was considered to have occurred de novo. According to the ACMG/AMP guidelines, the variant was classified as pathogenic. Brain MRI was performed at confirmation of central hypoventilation to rule out other differential diagnoses prior to the result of the genetic test [5]. This revealed a ‘quantitative reduction of the bihemispheric white matter, more pronounced in the posterior regions, with T2/FLAIR hyperintensities involving the peritrigonal regions and corona radiata, associated with dilated perivascular spaces, suggestive of possible gliosis’ (Figure 1). These findings were considered suggestive of hypoxic injury, potentially related to the child’s underlying respiratory condition, while they appear inconsistent with congenital abnormalities or abnormalities arising during the neonatal period, given the normality of the previous cranial ultrasound.

Additional investigations were conducted, including ophthalmological and neurological evaluations, electroencephalography (EEG), urinary catecholamine levels, abdominal ultrasound, fecal occult blood testing, Holter ECG, and echocardiography. All results were normal except for the EEG, which showed background activity with slowing over the central derivations, but no epileptiform abnormalities. Child neuropsychiatric evaluation (NPI) revealed a mild delay in achieving motor milestones and verbal language impairment; consequently, the patient was enrolled in a neurodevelopmental rehabilitation program. As recommended by the most recent guidelines [1], blood pressure was measured three times a day non-invasively using an appropriately sized arm cuff and revealed persistently elevated values, exceeding the 99th percentile for both systolic and diastolic pressure. There was no family history of hypertension. Antihypertensive therapy with amlodipine was initiated, but the clinical response was suboptimal. A renal Doppler ultrasound was performed to exclude renal artery stenosis as a secondary cause of hypertension. Endocrine causes of hypertension were also excluded based on normal thyroid function tests, serum cortisol, renin and aldosterone, and urinary vanillylmandelic acid (VMA), homovanillic acid (HVA), and metanephrines/normetanephrines. Gradual escalation of antihypertensive therapy with calcium channel blockers led to improved blood pressure control, with values consistently maintained between the 90th and 95th percentiles. There was no evidence of end-organ damage, and both fundoscopic examination and echocardiography were normal. Before discharge, home blood pressure monitoring was recommended, with a re-evaluation after one month. However, at the outpatient follow-up, blood pressure values were persistently elevated. This led to a change in antihypertensive therapy, with the addition of an angiotensin II receptor blocker (ARB). At the subsequent follow-up, blood pressure values were within the normal range.

## 3. Discussion

### 3.1. Diagnostic Considerations

A diagnosis of CCHS should be considered in infants or children presenting with unexplained hypercapnia, particularly when CO_2_ levels vary significantly between sleep and wakefulness. In this case, hypercapnia during sleep, rather than during wakefulness, was the key factor. Late-onset central hypoventilation is often triggered by respiratory infections, which increase respiratory demand. Therefore, capnography during sleep is essential for an accurate assessment. Genetic analysis of the *PHOX2B* gene is crucial to confirm CCHS and distinguish it from other causes of central hypoventilation, such as ROHHAD syndrome, familial dysautonomia, or syndromes in which central hypoventilation occurs secondarily, including Prader–Willi syndrome.

### 3.2. Genotype–Phenotype Relationship

The c.42C>A (p.Tyr14Ter) mutation, which introduces a premature stop codon at position 14, is located in exon 1 of the *PHOX2B* gene. In theory, such an early stop codon would typically trigger nonsense-mediated mRNA decay (NMD), preventing the production of a truncated protein. However, this does not appear to occur here. In “In Vitro Studies of non-Poly Alanine *PHOX2B* Mutations”, Trochet et al. [3] demonstrated that translation can resume downstream of the premature stop codon due to the presence of two alternative in-frame start codons (AUG18 and AUG21), potentially allowing the synthesis of a partially functional protein. This mechanism results in the production of a truncated *PHOX2B* protein that retains partial functionality. Immunocytochemistry studies confirmed expression of both wild-type and p.Tyr14Ter proteins in transfected HeLa cells, supporting the hypothesis that translation can reinitiate downstream of the premature stop codon.

### 3.3. Hypertension and Autonomic Dysfunction

**Figure 2:**
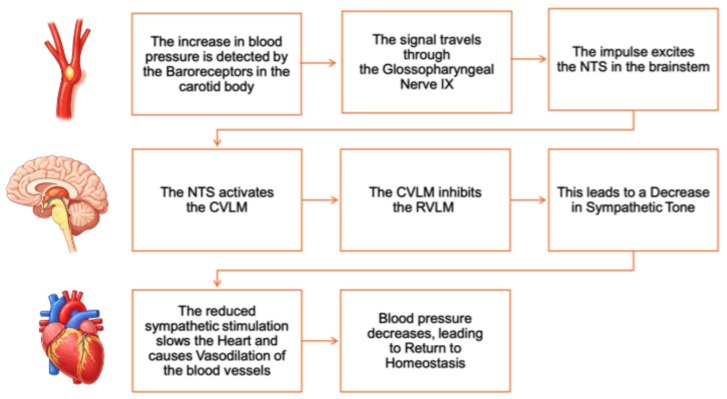
Schematic representation of the baroreflex pathway, from baroreceptor activation to the decrease in arterial blood pressure.

Both functional and anatomical studies have demonstrated baroreflex dysfunction in patients with CCHS. This is associated with abnormal development of the brainstem structures involved in autonomic regulation due to *PHOX2B* gene mutations. The baroreflex, also known as the baroreceptor reflex, is a key physiological mechanism for maintaining blood pressure homeostasis. It relies on baroreceptors located in the carotid sinus and the aortic arch. Rising blood pressure activates these baroreceptors, stimulating neurons in the nucleus of the solitary tract (NTS), which excite the caudal ventrolateral medulla (CVLM). The CVLM inhibits the rostral ventrolateral medulla (RVLM), which normally provides excitatory input to sympathetic preganglionic neurons; inhibition of the RVLM results in decreased sympathetic tone and consequently lower blood pressure. Conversely, when blood pressure drops, baroreceptor activity decreases, leading to the disinhibition of the RVLM and subsequently increasing blood pressure [6] (shown in Figure 2).

Baroreflex function has been investigated in patients with CCHS, with all studies consistently demonstrating various degrees of impairment. This dysfunction can lead to several effects, including failure of the sympathetic nervous system to adequately disinhibit during postural changes—such as from supine to upright—potentially resulting in orthostatic hypotension.

Trang et al. measured the blood pressure of 16-year-old subjects with a diagnosis of CCHS in supine, head-up tilt, and standing positions, finding normal values at rest. However, the maneuvers demonstrated a limited capacity to increase blood pressure appropriately in response to orthostatic stress, indicating impaired autonomic compensation [7].

Eric L. Vu et al. evaluated blood pressure values in subjects with CCHS and control subjects during head-up tilt testing. During tilt-up, subjects with CCHS experienced a greater decrease in mean blood pressure than control subjects [8].

Impaired baroreflex function during sleep may also contribute to the absence of physiological nocturnal blood pressure dipping and, in some cases, nocturnal hypertension in patients with CCHS. A retrospective study enrolled children with CCHS and monitored their blood pressure over 24 h. The prevalence of hypertension (33%) was higher than expected. CCHS genotype influenced elevated blood pressure and hypertension rates, with higher prevalence in those with severe polyalanine repeat mutations (PARMs) versus NPARM genotypes. Unlike our patient, who carries a non-polyalanine repeat mutation (NPARM), the aforementioned study did not report any blood pressure abnormalities in the non-PARM group [9]. This observation is in line with other reports of patients carrying an NPARM, as detailed in Table 1 and in a recent study aiming to define genotype–phenotype correlation in NPARM patients [10].

These results highlight variability in autonomic dysfunction expression, even within the same mutation category. Another study demonstrated a significant reduction in carotid body glomus cells in CCHS patients, and ultrastructural analysis revealed marked alterations in these cells, supporting the concept of deficient ‘hardware’ in CCHS [16].

## 4. Conclusions

This case report describes a de novo *PHOX2B* nonsense mutation associated with congenital central hypoventilation syndrome, presenting in infancy with sleep-related hypercapnia. The unusual finding of early-onset hypertension suggests significant autonomic dysregulation, likely related to impaired baroreflex control.

These findings underscore the importance of routine blood pressure monitoring in all CCHS patients, regardless of *PHOX2B* mutation type. Further studies are needed to establish long-term monitoring and management strategies for cardiovascular complications in this population. The subtle phenotype of this mutation may also explain the delayed diagnosis in our patient, despite early signs like neonatal respiratory distress.

This highlights CCHS phenotypic variability and the importance of early diagnosis in cases of unexplained hypercapnia or neonatal respiratory distress. Further studies are needed to clarify the genotype–phenotype correlation in rare *PHOX2B* mutations.

## Figures and Tables

**Table 1:** Early truncating variants (NPARMs) in *PHOX2B* previously reported as case reports and their related clinical phenotypes. All reported variants occurred in exon 1 and were nonsense mutations. None of the cited case reports included the results of blood pressure monitoring.

Variant	Clinical Data	Specific Findings About Blood Pressure or Dysautonomy
c.13G>T (p.Glu5*) [11]	Neonatal respiratory failure without respiratory distress; at two-years, only non-invasive ventilation (NIV) during sleep	Not reported
c.18T>C (p.Tyr6*) [11]	Cyanotic spells from early infancy	Constipation Normal ECG and Holter
c.18T>G (p.Tyr6*) [12]	No clinical data	No clinical data
c.23dupA (p.Tyr8*) [13]	Three episodes of viral-induced type II respiratory failure. Subsequent diagnosis and NIV during sleep. Isolated hypoventilation on polysomnography (PSG) with no apneas.	Normal ECG, Holter, and echocardiography
c.42C>A (p.Tyr14*) [3]	CCHS identified at 5 weeks of age.	No information
c.234C>G [14]	Long-segment colonic aganglionosis Congenital heart disease in two of the three affected patients	Anisocoria
c.234C>G [15]	Two-month-old female with birth hypoglycemia, hypercarbia, stridor, gastro-esophageal reflux disease, and laryngomalacia. PSG showed severe OSA and hypoventilation. The patient required tracheostomy	No cardiac arrhythmias

## Data Availability

The data supporting the findings of this case report are available from the corresponding author upon reasonable request, in accordance with ethical and privacy restrictions.

## Abbreviations

CCHS	Congenital Central Hypoventilation Syndrome
PICU	Pediatric Intensive Care Unit
RSV	Respiratory Syncytial Virus
PARMs	Polyalanine repeat mutations
CVLM	Caudal ventrolateral medulla
RVLM	Rostral ventrolateral medulla
